# Supplementary material for: MetaQTL: a package of new computational methods for the meta-analysis of QTL mapping experiments
Source: BMC Bioinformatics. 2007 Feb 8;8:49. doi: 10.1186/1471-2105-8-49 (PMC1808479; doi:10.1186/1471-2105-8-49)
Supplement: Additional File 7 — MetaQTL Package : jar file and tutorial. This Zip archive contains both the MetaQTL JAR file and the files of the tutorial. [file 1471-2105-8-49-S7.zip › org.inra.metaqtl/doc/org/metaqtl/MultiFitResult.html]

MultiFitResult


|  |  |  |
| --- | --- | --- |
| **Overview** \| **Package** \| **Class** \| **Use** \| **Tree** \| **Deprecated** \| **Index** \| **Help** | |  |
| **PREV CLASS**   **NEXT CLASS** | **FRAMES**    **NO FRAMES**     **All Classes** |  |
| SUMMARY: NESTED \| FIELD \| CONSTR \| METHOD | DETAIL: FIELD \| CONSTR \| METHOD |  |


---


## org.metaqtl Class MultiFitResult

```
java.lang.Object
  org.metaqtl.MultiFitResult
```

---

``` public class MultiFitResult extends java.lang.Object ```

This class represents a result of a multilinear regression
analysis.

---

| **Field Summary** | |
| --- | --- |
| `double` | `chi2`             The chi-square of the model. |
| `double[][]` | `cov`             The variance-covariance matrix of the parameter estimates. |
| `int` | `dof`             The number of degrees of freedom. |
| `int` | `m`             The number of parameters |
| `int` | `n`             The number of observations without missing data. |
| `double` | `pvalue`             The p-value of the model. |
| `double[]` | `rsd`             The residual standard deviations. |
| `double[]` | `theta`             The parameter estimates. |


**Constructor Summary**

`MultiFitResult(int N,
int M)`
  
          Creates a new instance of `MultiFitResult`
with the given number of observations `N`
and parameters `M`.


| **Method Summary** | |
| --- | --- |
| `java.lang.String` | `toString()` |

**Methods inherited from class java.lang.Object**

`clone, equals, finalize, getClass, hashCode, notify, notifyAll, wait, wait, wait`

**Field Detail**

### pvalue

```
public double pvalue
```

:   The p-value of the model.

---


### theta

```
public double[] theta
```

:   The parameter estimates.

---


### cov

```
public double[][] cov
```

:   The variance-covariance matrix of the parameter
    estimates.

---


### chi2

```
public double chi2
```

:   The chi-square of the model.

---


### rsd

```
public double[] rsd
```

:   The residual standard deviations.

---


### m

```
public int m
```

:   The number of parameters

---


### n

```
public int n
```

:   The number of observations without missing data.

---


### dof

```
public int dof
```

:   The number of degrees of freedom.


**Constructor Detail**

### MultiFitResult

```
public MultiFitResult(int N,
                      int M)
```

:   Creates a new instance of `MultiFitResult`
    with the given number of observations `N`
    and parameters `M`.

    **Parameters:**: `N` - the number of observations.: `M` - the number of parameters.


**Method Detail**

### toString

```
public java.lang.String toString()
```

:   **Overrides:**: `toString` in class `java.lang.Object`


---


|  |  |  |
| --- | --- | --- |
| **Overview** \| **Package** \| **Class** \| **Use** \| **Tree** \| **Deprecated** \| **Index** \| **Help** | |  |
| **PREV CLASS**   **NEXT CLASS** | **FRAMES**    **NO FRAMES**     **All Classes** |  |
| SUMMARY: NESTED \| FIELD \| CONSTR \| METHOD | DETAIL: FIELD \| CONSTR \| METHOD |  |


---
